# Kinetic Modeling and Numerical Simulation as Tools to Scale Microalgae Cell Membrane Permeabilization by Means of Pulsed Electric Fields (PEF) From Lab to Pilot Plants

**DOI:** 10.3389/fbioe.2020.00209

**Published:** 2020-03-24

**Authors:** Justus Knappert, Christopher McHardy, Cornelia Rauh

**Affiliations:** Department of Food Biotechnology and Food Process Engineering, Technische Universität Berlin, Berlin, Germany

**Keywords:** Pulsed Electric Fields, numerical simulation, computational fluid dynamics, microalgae, inactivation kinetic, scale- up, cell membrane permeabilization

## Abstract

Pulsed Electric Fields (PEF) is a promising technology for the gentle and energy efficient disruption of microalgae cells such as *Chlorella vulgaris*. The technology is based on the exposure of cells to a high voltage electric field, which causes the permeabilization of the cell membrane. Due to the dependency of the effective treatment conditions on the specific design of the treatment chamber, it is difficult to compare data obtained in different chambers or at different scales, e.g., lab or pilot scale. This problem can be overcome by the help of numerical simulation since it enables the accessibility to the local treatment conditions (electric field strength, temperature, flow field) inside a treatment chamber. To date, no kinetic models for the cell membrane permeabilization of microalgae are available what makes it difficult to decide if and in what extent local treatment conditions have an impact on the permeabilization. Therefore, a kinetic model for the perforation of microalgae cells of the species *Chlorella vulgaris* was developed in the present work. The model describes the fraction of perforated cells as a function of the electric field strength, the temperature and the treatment time by using data which were obtained in a milliliter scale batchwise treatment chamber. Thereafter, the model was implemented in a CFD simulation of a pilot-scale continuous treatment chamber with colinear electrode arrangement. The numerical results were compared to experimental measurements of cell permeabilization in a similar continuous treatment chamber. The predicted values and the experimental data agree reasonably well what demonstrates the validity of the proposed model. Therefore, it can be applied to any possible treatment chamber geometry and can be used as a tool for scaling cell permeabilization of microalgae by means of PEF from lab to pilot scale. The present work provides the first contribution showing the applicability of kinetic modeling and numerical simulation for designing PEF processes for the purpose of biorefining microalgae biomass. This can help to develop new processes and to reduce the costs for the development of new treatment chamber designs.

## Introduction

Microalgae are often considered to have a great potential as a resource for the biotechnology and food industries. Due to the high protein content in some species of up to 70% of the dry weight (Becker, 2007), they are a promising alternative source for high value proteins. These are particular needed in the near future since it is expected that the world population reaches 9.8 billion people in 2050 (United Nations- Department of Economic and Social Affairs, 2017). Besides proteins, microalgae can deliver many other ingredients with health benefits such as polyunsaturated fatty acids (PUFAs), pigments or diverse carbohydrates, what makes them even more interesting for applications in the food industry (Caporgno and Mathys, 2018). But except from some niche products, such as high valuable pigments or whole cells as food additives, microalgae did not reach marked maturity today (Lam et al., 2018). This is attributed to the high cost of production for microalgae biomass and their intracellular metabolites. A major part of the production costs is associated with the downstream processing, which includes all processing steps after the cell cultivation, and which is crucial for the production of valuable metabolites. A key process thereby is the disruption of the cells which enhances or even enables the extractability of almost all intracellular metabolites (Ruiz et al., 2016). Except of water-soluble substances such as some proteins or carbohydrates, the extraction of valuables like pigments or fatty acids requires the use of non-polar solvents. However, many green solvents such as ethanol cannot penetrate the cells and therefore, they do not reach the same yield as other solvents such as chloroform, hexane or butanol (Zbinden et al., 2013), which on the other hand have a negative impact on the environment, health and safety (Capello et al., 2007) and are therefore not approved for applications in the food and beverage industry. Cell disruption is therefore indispensable to enable the use of green solvents such as ethanol and moreover, it is essential for using algae biomass as a raw material within a biorefinery concept.

Because some species are characterized by a small cell size of a few micrometers and a rigid cell wall, the disruption of microalgae cells as *Chlorella* needs a high amount of energy and therefore, the recovery of valuables from microalgae cells becomes very expensive. Today, there are several mechanical techniques available for cell disruption such as bead milling (BM), high pressure homogenization (HPH), ultrasonication (US), microwave treatment (MW) or Pulsed Electric Field (PEF) (Günerken et al., 2015). Even if high pressure homogenization and bead milling are considered to be the most effective techniques for cell disruption, their main disadvantage is the resulting small cell debris and the non-selective release of intracellular components (D’Hondt et al., 2017). A promising alternative is disruption of cells by means of Pulsed Electric Fields (PEF). The technique is based on the exposure of cells to an external pulsating electric field which leads to an uprising transmembrane potential. The formation of hydrophilic pores in the cell membrane is favored if the transmembrane potential exceeds a critical value (Glaser et al., 1988). Consequently, the cell membrane is permeabilized, what leads to the enhancement of mass transfer processes across the cell membrane (Pataro et al., 2017). Because of this mechanism, the attention for applying PEF to improve the extraction of metabolites from microalgae cells increased in the last years. The target molecules contained a large variety of valuables, namely pigments like chlorophyll, lutein or phycocyanin (Grimi et al., 2014; Luengo et al., 2014, 2015; Parniakov et al., 2015; Zocher et al., 2016; Martínez et al., 2017), carbohydrates (Parniakov et al., 2015; Postma et al., 2016; Pataro et al., 2017; Carullo et al., 2018), proteins (Grimi et al., 2014; Aouir et al., 2015; Coustets et al., 2015; Parniakov et al., 2015; Postma et al., 2016; Lam et al., 2017a, b; Nehmé et al., 2017; Pataro et al., 2017), and lipids (Goettel et al., 2013; Zbinden et al., 2013; Silve et al., 2018a, b). All these studies have in common that the work was done at a microliter or milliliter scale and that the achieved results are specific for the used equipment and the respective treatment homogeneity. Because the distribution of an electric field inside PEF treatment chambers strongly depends on the geometry, it is difficult to compare the results of the listed works and furthermore, it is nearly impossible to scale the results to a plant at larger scale.

Until today there is no valid tool to transfer results obtained in PEF plants at laboratory scale to plants at the pilot or even the industrial scale. The strong dependency of the treatment homogeneity, namely the electric field distribution, temperature distribution and the velocity field, on the geometry of the treatment chamber has been shown by many numerical investigations (Toepfl et al., 2007; Gerlach et al., 2008; Jaeger et al., 2009; Rauh et al., 2010; Meneses et al., 2011). For example, Gerlach et al. demonstrated the dependency of the electric field homogeneity on the inner radius of the insulator between the high voltage and the grounding electrodes of a colinear treatment chamber. But even if the electric field, temperature and velocity distributions inside such a chamber are known, a direct evaluation of the impact of local conditions on the overall degree of cell permeabilization is difficult if permeabilization itself is not simulated as well.

In order to evaluate such effects numerically, a model is required which describes the rate of cell permeabilization as a function of the electric field strength, temperature magnitude and exposure time. Such a model can be included in numerical simulations of the PEF process so that the overall degree of cell permeabilization can be evaluated as the summarized effect of the local treatment conditions on the cell population while passing the treatment zone. However, no suitable model for the effect of PEF on microalgae is available so far. This gap shall be closed with the present work. The objective of this study is therefore to derive a kinetic model for the cell permeabilization of the microalgae *Chlorella vulgaris* from data gained in laboratory experiments being conducted on a small cuvette scale with homogeneous treatment conditions. The kinetic model is thereafter implemented in the commercial software Ansys CFX19 as part of a holistic numerical model for the PEF process in order to investigate by numerical simulation the effects of different treatment conditions inside a colinear treatment chamber with a volume flow of up to 15 L h^−1^. Furthermore, experiments are conducted in a similar setup in order to show that the kinetic model is capable of correctly predicting the overall degree of cell permeabilization. If this condition is met, it can be a useful tool for designing treatment chambers on the industrial scale on the basis of data from laboratory experiments.

## Materials and Methods

### Microalgae Cultivation

All experiments were performed using *Chlorella vulgaris*, strain number SAG 211-11b, obtained from the algae collection from the University of Göttingen (SAG, Culture Collection of Algae, Göttingen, Germany). The strain was maintained until its usage in Erddekot + Salz + Peptone-medium (ESP-Medium) under continuous illumination of 20 μmol m^–2^ s^–1^ on a shaker (100 rpm). The recipe for the medium is provided by the SAG.

To produce biomass for the experiments, *Chlorella vulgaris* was cultivated autotrophically in a bubble column photobioreactor with a culture volume of 1.5 L (reactor volume 1.65 L). The cultivation was carried out in modified Bolds Basal Medium with threefold nitrogen and Vitamins (3N-BBM + V medium), according to the recipe of the Culture Collection of Algae and Protozoa. The culture was aerated with air enriched with 2.5% CO_2_ at a volume flow rate of 800 ml min^–1^. Artificial illumination was realized with two LED panels (LED-Mg) with a total area of 0.171 m^2^. The light intensity was set to 150 μmol m^–2^ s^–1^, which was measured on a fixed point of reference in the middle of the bubble column reactor right above the liquid level. The whole bubble column was placed in a tempered water bath to control the temperature at 25°C. The cells were harvested semi-continuously every third day via a sampling port. The volume of harvested cell suspension was replaced with autoclaved 3N-BBM + V medium. A preliminary test was carried out to determine the specific growth rate of the culture in order to make sure that it is always in the late exponential growth phase when harvested (data not shown).

To determine the cell density of the culture, a correlation between the biomass dry weight and the extinction at 750 nm was established. The extinction of several diluted cell samples was measured in 1 cm cuvettes by using an UV/VIS-Spectrometer (Lambda 25, Perkin Elmer). The cell dry weight of the same samples was determined by filtering through pre-dried glass microfiber filters (Whatman GF/F, pore size 0.7 μm). After filtering the samples, the filters were washed twice with distilled water to remove salts and then dried for 24 h at 80°C. For each data point, cell dry weight and extinction were measured in triplicate.

### PEF Treatment

#### Sample Preparation

After collecting the cells, the dry biomass concentration for all PEF treatments was adjusted to 1 g L^–1^. Therefore, the microalgae suspension was concentrated by centrifugation at 5000 g for 10 min before adjusting the cell concentration. After centrifugation, the pellet was resuspended in fresh 3N-BBM + V medium so that the desired cell concentration resulted, which was verified by UV/VIS Spectrometry. The cell suspension was tempered to the specific temperature of the respective experiment in 2 ml tubes (Eppendorf, Germany) on a shaker for a minimum of 15 min. 3N-BBM + V medium served as the treatment media. Its electrical conductivity at 20°C was 1.3 mS cm^–1^.

#### PEF Systems and Experimental Design

Pulsed Electric Fields-treatments at cuvette scale were performed in order to derive data for the kinetic modeling. The experiments were conducted in a prototype treatment plant, being designed inhouse. The storage capacity of the plant is 19.5 nF [Ceramite Y5U 6800Z-Kondensatoren (Behlke Electronic GmbH, Kronberg, Germany)]. It is charged by a 20 kV and 80 mA high voltage charging unit. The capacitors are discharged in exponential decay pulses using a thyristor switch (HTS 160-500SCR, Behlke Electronic GmbH, Kronberg, Germany). Voltage and current were directly measured at the treatment chamber by a 75 MHz high voltage probe and a 100 MHz current probe, respectively. The signals were visualized with a 400 MHz digital storage oscilloscope (TDS220-Oszilloskop, Sony Tektronix, Beaverton, United States). Electroporation cuvettes (VWR) with parallel plates and an electrode gap of 4 mm were used for the treatment. Prior to each experiment, the electroporation cuvettes were tempered to the desired treatment temperature in a sand bath for at least 30 min. For temperature control during the experiment, the whole plant was placed below a temperature-controlled incubator hood (Certomat, HK, Sartorius, Germany). The effect of electric field strength, initial treatment temperature and treatment time was investigated. Therefore, the electric field strength was varied in 5 steps, namely 6.5, 9, 13.5, 20, and 27 kV cm^–1^. The effect of the temperature was investigated on three stages at 20, 30, and 40°C for every level of the applied electric field strength. For all combinations of temperature and electric field strength, the cells were treated with 0, 1, 2, 4, 8, 16, 32, and 64 exponential electric pulses with a time constant of 3.7 ± 0.63 μs, which measures the time in which the pulse decays to 64% of the peak voltage. The temperature in the electroporation cuvettes was measured directly before and after the treatment with a manual measuring device. The highest measured temperature increase was 8.2°C. All experiments were conducted in duplicate and those with 64 pulses in triplicate.

**FIGURE 1:**
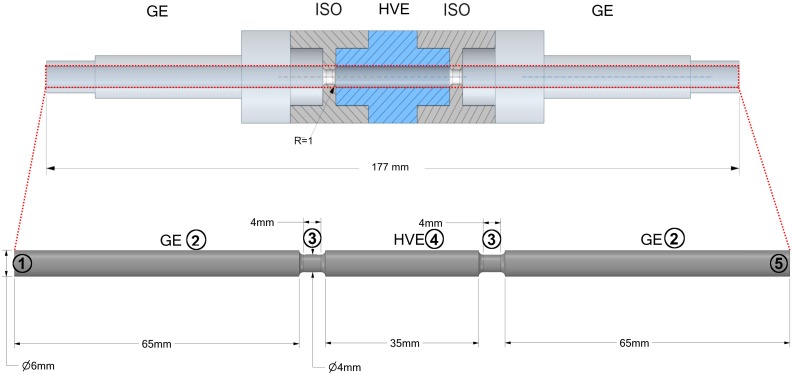
Schematic view of a colinear treatment chamber as it was used for CFD simulations and pilot scale experiments. The dash-dotted line indicates the symmetry of the domain, whereby the outlet is located at the right end of the fluid domain. The numbers indicate the boundaries of the domain for which the boundary conditions are summarized in Table 2. GE, Grounding electrode; ISO, Insulator; HVE, High voltage electrode.

A second series of experiments was conducted in a treatment plant at pilot scale with a colinear treatment chamber. The assembly of the treatment chamber is the same as the one being investigated in numerical simulations (see Figure 1). The inner diameter of the electrodes and the insulators was 6 mm and 4 mm, respectively. The length of the grounding and high voltage electrode was 65 mm and 35 mm, respectively. The length of the insulators was 4 mm. Both, the high voltage and the grounding electrodes are manufactured from stainless steel. A 7 kW modulator (ScandiNova) was used to deliver quasi-rectangular monopolar pulses with a pulse width of 5 μ*s*. An overshoot and ringing of the voltage was observed during the pulse, which is caused by the electrical conductivity of the 3N-BBM + V medium and the related impedance mismatching. Despite this observation, the electrical conductivity of the medium was not changed with regard to the industrial application of PEF in the context of microalgae biorefinery and the respective typical composition of growth media. The *C. vulgaris* cell suspension was pumped through the entire system by using a micro annular gear pump (HNP Mikrosysteme, Germany, type: mzr 7205). Before entering the treatment chamber, the cell suspension was tempered to the treatment temperature in a heat exchanger. In order to drive the system into a thermal steady-state (no further heating of pipes and electrodes), a saline solution with the same conductivity as the microalgae suspension was treated prior to each experiment with similar conditions. The achievement of steady-state conditions was monitored by measuring the temperature of the liquid before and after the treatment chamber. After reaching constant conditions, a three-way valve was used to switch between the saline solution and the microalgae suspension. The residence time inside the whole plant was determined in a preliminary experiment using food colors (data not shown). In accordance to these measurements, samples of the treated algae were not taken before a time interval of 2 min has been passed after switching from saline solution to algae suspension. For each parameter set two samples were taken within a time interval of at least 30 s. After taking the samples, the valve was switched back to the saline solution, the pulse generator was switched off and the next treatment parameters were set. According to the experimental plan, the liquid volume flow was varied between 100 and 200 ml min^–1^, the inlet temperature from 30 to 40°C and the applied voltage from 7 to 15 kV, being measured directly at the treatment chamber. To exclude possible effects on the measured degree of cell disruption by preheating or by the pump, a zero sample was taken before switching the pulse modulator on.

#### Flow Cytometry

The fraction of perforated cells (*F*_*p*_) in all treated samples was determined by the uptake of the fluorescents dye propidium iodide (PI, concentration *c* = 1000 mg ml^–1^; Molecular Probes; Lot No.: 45B5; Exmax/Emmax 492/638). PI is a hydrophilic dye which cannot penetrate through the intact cell membrane. After the PEF treatment, cell suspensions were directly diluted in ice cooled 3N-BBM + V medium and stored on ice until further use. The dilution factor was 1:100 which leads to a dry biomass concentration of around 10 mg L^–1^ or 10^6^ cells ml^–1^, respectively (checked with a hemocytometer). The PI was added 15 ± 2 min after the PEF treatment so that only the irreversibly perforated cells were detected with this staining protocol (Luengo et al., 2014). PI was added to a final concentration of 11 μg ml^–1^. The samples were shaken in the dark at 23°C and 300 rpm for 15 min. The fluorescent signal was measured with a flow cytometer (BD Accuri C6, Becton Dickinson, United States). The signal was captured on the FL2 emission filter at 585/40 nm. The flow rate was set to 14 μl min^–1^ and 20 μl per sample were measured at a front scatter threshold of 80000 (arbitrary units). A preliminary test was conducted for the distinction between the signal of the perforated and the unperforated cells. Therefore, a mixture of 50% living algae cells and of 50% heat inactivated cells was prepared. Two clearly separable peaks occurred, and each peak contained 50% of the overall measured events, whereby the peak with the stronger fluorescence signal represented the perforated cells. A line of calibration was inserted between these peaks and was saved as a template for further experiments. Each treated sample was stained in duplicates and all stained samples were measured as duplicates.

### Numerical Model

#### Governing Equations

The governing equations are based on the conservation equations for mass, momentum, energy and charge (Wölken et al., 2017; Fiala et al., 2001; Lindgren et al., 2002; Gerlach et al., 2008; Jaeger et al., 2009). Specific assumptions applied in this work are that the presence of cells does not affect the flow of the suspension and therefore, the cell suspension can be treated as an incompressible single-phase fluid. These assumptions can be justified with the low concentration of cells during the treatment (1 gL^−1^) and their small size, leading to a low volume fraction, negligible cell-cell interactions and a small value of the Stokes number (Crowe et al., 2011). Then, the conservation equation for mass can be written as

(1) ∇⋅u=0

Where *u* is the fluid velocity vector. It is assumed that no direct effects of the electric field on the flow exist. Therefore, the conservation equation for momentum is given by

(2) $$\frac{\partial \rho \,\text{u}}{\partial t}+\nabla \cdot \left(\rho \,{\text{uu}}^{T}\right)=-\nabla p+\nabla \cdot \mu \,\left(\nabla \text{u}+{\left(\nabla \text{u}\right)}^{\text{T}}\right)+\rho \,\text{g}$$

Wherein *t* is the time, ρ is the fluid density, *p* is the pressure, μ the fluid dynamic viscosity and *g* the vector of gravitational acceleration. Because of the incompressibility of the fluid, its internal energy can be described by the applied thermal energy alone. Therefore, the energy conservation equation becomes

(3) $$c_{p}\,\frac{\partial \rho \,T}{\partial t}+c_{p}\,\nabla \cdot \left(\rho \,\text{u}\,T\right)=\nabla \cdot \left(\lambda \,\nabla T\right)+\pi_{e}$$

wherein *c*_*p*_ is the heat capacity of the fluid, *T* the total temperature and *K* the fluids thermal conductivity. The term π_*e*_ represents a source term for the internal energy. In the case of PEF, the source term describes the increase of internal energy by Joule heating. The thermal transport only takes place during the duration τ of a pulse so that the source term must be corrected by the effective fraction of time during which the electric field is active, which is given by the product of the pulse duration and the pulse repetition rate *f*. Thus, the source term in equation (3) becomes

(4) $$\pi_{e}=\tau \,f\,\sigma \,E^{2}$$

Where σ is the electric conductivity of the fluid and *E* represents the local strength of the electric field. For the computation of the latter, another equation is needed. The electric field strength can be calculated by solving a transport equation for carriers of electric charge. With the assumptions of an electrostatic field, charge conservation and Ohm’s law for the electric current, the transport equation reduces to a Laplace equation for the scalar electric potential Φ (Wölken et al., 2017):

(5) ∇⋅(σ⁢∇⁡Φ)=0

By solving (5), the electric field can be computed from the electric potential with the relation:

(6) E=-∇⁡Φ

The fraction *F*_*p*_ of permeabilized microalgae cells is a local property of the carrier cell population, which is affected by the action of the treatment and its transport in space. The transport of *F*_*p*_ is coupled to the transport of the cells. As mentioned before, microalgae cells are considered as passive tracers which have no influence on the fluid behavior and do not interact with each other. This is a safe assumption at microorganism concentrations in the order of 10^14^ m^–3^ as being considered here and it implies that the transport of cells by diffusion is not significant. Thus, the transport equation for the activity of passive biological tracers *F*_*p*_ can be expressed as follows (Rauh et al., 2009):

(7) $$\frac{\partial F_{p}}{\partial t}+\nabla \cdot \left(u\,F_{p}\right)=\pi_{{\text{F}}_{\text{p}}}$$

The term π_*F_p*_ represents a source term for the fraction of perforated cells. It is a function of the electric field strength and the treatment temperature and will be derived in section “Results” from experimental data.

#### Thermophysical Fluid Properties

The material properties of the fluid depend strongly on the temperature. The temperature dependency of the material properties is therefore considered according to the equations being listed in Table 1.

**TABLE 1:** Temperature dependency of the fluid material properties.

**Property**	**Symbol**	**Equation**
Density^1^	ρ[*kg*^m−3^]	$1000.22+1.0205\cdot 10^{-2}\cdot T_{c}-5.8149\cdot 10^{-3}\cdot T_{c}^{2}+1.496\cdot 10^{-5}\cdot T_{c}^{3}$
Heat capacity^1^	*c*_*p*_[*kJkg*^−1^^K−1^]	$4176.2-0.0909\cdot T_{c}+5.4731\cdot 10^{-3}\cdot T_{c}^{2}$
Thermal conductivity^1^	κ[W^m−1^^K−1^]	$0.57109+1.7625\cdot 10^{-3}\cdot T_{c}-6.7036\cdot 10^{-6}\cdot T_{c}^{2}$
Dynamic viscosity^1^	μ[*kg*^m−1^^s−1^]	$2.414\cdot 10^{-5}\cdot 10^{\left(\frac{247.8\,\left[K\right]}{T_{K}-140\,\left[K\right]}\right)}$
Electric conductivity^2^	σ[S^m−1^]	$0.889\cdot 10^{\frac{A}{B}}\cdot \sigma_{r\,e\,f}$
	*A*	1.37023⋅(Tc-20⁢C∘)+8.36⋅10-4⋅(Tc-20⁢C∘)2
	*B*	109 + *T*_*c*_
	σ_*ref*_	1.2 *mScm*^−1^

*The subscript C describes the temperature in degree Celsius, the subscript K stands for Kelvin. ^1^Wölken et al., 2017; ^2^Atkins and de Paula, 2006.*

#### Computational Domain and Grid Generation

The computational domain as depicted in Figure 1 consists of two grounding electrodes (2), one high voltage electrode (4) and two isolator rings (3). The numbers (1) and (5) indicate the inlet and the outlet of the fluid domain, respectively. The domain was automatically discretized with tetrahedral mesh elements by means of the software Ansys Meshing. The mesh was additionally refined in regions where high gradients of the flow velocity were expected, especially within and after the isolator rings. Also, the region at the wall of the domain was meshed by prism layers since a large gradient of the velocity can be expected here due to the no slip boundary condition. A mesh convergence study with 5 different meshes was performed in which the effect of the mesh element size on *F*_*p*_ and the flow and temperature fields was studied. The final mesh was chosen such that the deviation of all quantities was less than 3% in relation to their value on the finest of all generated meshes.

#### Boundary Conditions

The applied boundary conditions are summarized in Table 2. For the inflow, a parabolic velocity profile was assumed according to expectable laminar flow conditions, which were estimated by means of the Reynolds number. The inlet velocity profile was calculated from the Hagen-Poiseuille equation for a given volumetric flow rate $\dot{V}$. At the walls, the common no-slip condition was assumed, thus the liquid velocity is zero. At the inlet of the chamber, a static temperature *T*_*0*_ was assumed. Furthermore, adiabatic walls were assumed, thus no heat flux across the walls. This assumption is justified because PEF treatment chambers are usually covered by insulating materials. For the electrostatic model zero-flux boundary conditions at the inlet, outlet and the isolators were assumed. The voltage at the high voltage electrode was set to a static value *U*_*0*_, while it was set to zero at the grounding. The fraction of perforated cells *F*_*p*_ was set to 0 at the inlet of the chamber and a zero-gradient condition was applied at the outlet.

**TABLE 2:** Boundary conditions applied in the CFD simulation.

**Location**	**Electrostatic model**	**Flow model**	**Thermal model**	**Transport equation**
Inlet (1)	∇⁡Φ = 0	$u=2\cdot \frac{\bar{V}}{\pi \,R^{2}}\cdot \left(1-{\left(\frac{r}{R}\right)}^{2}\right)$ *v=0 w=0*	*T* = *T*_0_	*F*_*p*_ = 0
Grounding (2)	*U=0*	*u*_*wall*_ = 0	*q*_*wall*_ = 0	∇⁡*F*_*p*_ = 0
Isolator (3)	∇⁡Φ = 0	*u*_*wall*_ = 0	*q*_*wall*_ = 0	∇⁡*F*_*p*_ = 0
High voltage electrode (4)	*U* = *U*_0_	*u*_*wall*_ = 0	*q*_*wall*_ = 0	∇⁡*F*_*p*_ = 0
Outlet (5)	∇⁡Φ = 0	*p*_*stat*_ = *p*_*spec*_	∇⁡*T* = 0	∇⁡*F*_*p*_ = 0

*The respective boundaries are indicated by the numbers in Figure 1.*

### Numerical Parameter Study

The numerical model described in section “Numerical Model” was used to perform a parameter study in order to investigate the effect of different treatment conditions on the degree of cell permeabilization. The specific parameter combinations were chosen by means of the response surface methodology. Flow rate, pulse repetition frequency, voltage and inlet temperature were chosen as explanatory variables and varied on 3 levels, whereby the levels of the voltage, flow rate, temperature and frequency were the same as applied in the second experimental series as described in section “PEF Systems and Experimental Design.” The respective levels are summarized in Table 3. Pulse duration was set to a fixed value of 5 μs. The degree of cell permeabilization and the temperature increase were chosen as response variables. The design of experiments led to 30 design points in total, whereby the repetitions were neglected because no variance occurs in numerical simulations with similar settings.

**TABLE 3:** Chosen parameter levels for the CFD parameter study.

	**Voltage [kV]**	**Inlet temperature [K]**	**Frequency [Hz]**	**Flow rate [*m*^3^*s*^−1^]**
Level 1	7	293	100	1.66e-06
Level 2	11	298	150	2.50e-06
Level 3	15	303	200	3.33e-06

## Results

### Experiments in Electroporation Cuvettes

**FIGURE 2:**
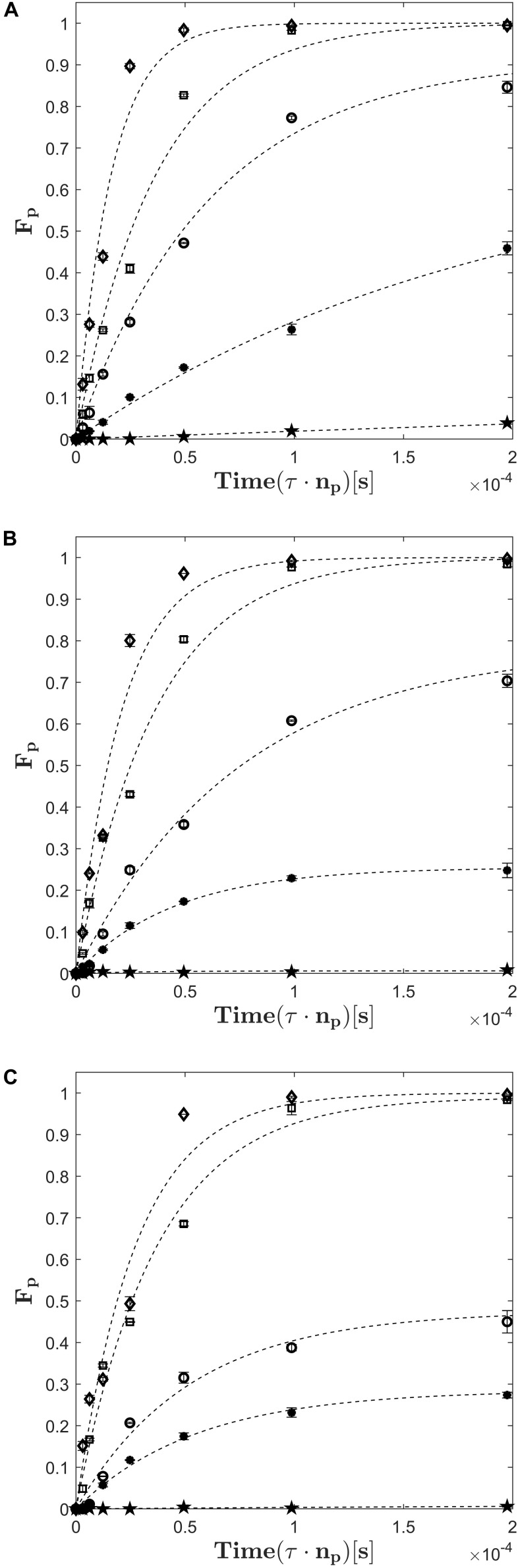
Fraction of perforated cells (*F*_*p*_) as a function of electric field strength, treatment time and treatment temperature (**A:** 40°C, **B:** 30°C, **C:** 20°C). The electric field strength is indicated by different symbols: 6.5*kVcm*^−1^ (stars), 9*kVcm*^−1^ (filled circles), 13.5*kVcm*^−1^ (empty circles), 20*kVcm*^−1^ (squares), 27*kVcm*^−1^ (diamonds). Error bars indicate the standard deviation. The dashed lines are best fits obtained with equation (9).

In order to obtain experimental data for the derivation of a kinetic model for cell perforation, experiments in electroporation cuvettes were conducted. The evolution of the fraction of perforated cells (*F*_*p*_) with respect to the treatment time, the electric field strength and the treatment temperature is presented in Figure 2. It can be seen that only treatments with electric field strength higher than 20 kV cm^–1^ led to a full perforation of the cell population at all tested treatment temperatures. Furthermore, Figure 2 shows that the increase of the electric field strength from 20 to 27 kV cm^–1^ reduced the treatment time being necessary for a complete perforation. For example, at 40°C and 27 kV cm^–1^, 100% of the cell suspension were permeabilized within a time of 0.48⋅10^−4^ s while at 40°C and 20 kV cm^–1^ twice the time was needed to achieve the same result. Note that the treatment time is the effective time during which the electric field is active.

An important parameter for assessing the electroporation process is the specific energy input *w*_*spec*_. For a batch treatment it can be calculated with the expression

(8) $$w_{s\,p\,e\,c}=\frac{U_{0}^{2}\,C\,n_{p}}{2\,m}$$

Here, *U*_*0*_ is the applied voltage (measured at the treatment chamber), *C* is the capacitance of the capacitors of the PEF plant, *n*_*p*_ is the number of applied pulses and *m* is the mass of the treated sample. According to equation (8), a specific energy input of 19.6 kJ kg^–1^ was needed to perforate 100% of the cells at 40°C and 27 kV cm^–1^, whereas 23.56 kJ kg^–1^ was necessary at 40°C and 20 kV cm^–1^. Even if these results might be biased because of the large difference between the applied treatment times, they indicate that the required specific energy input for the complete cell perforation decreases if the electric field strength increases. It seems therefore preferable to operate at high electric field strength in the case of industrial applications. Another result of the experiments is that the lowest applied field strength of 6.5 kV cm^–1^ was not high enough to perforate a considerable amount of *C. vulgaris* cells, see Figure 2. Even at the highest treatment temperature of 40°C, only around 4% of the population was perforated after the longest treatment time. Figure 2 further indicates that the treatment at 13.5 kV cm^–1^ and 9 kV cm^–1^ were insufficient to perforate the complete cell population, independently from temperature. Also, the fraction of perforated cells *F*_*p*_ converges to a maximum value (*F*_*p,max*_) for electric field strengths at which no complete perforation was achieved. For example, at 9 kV cm^–1^ and 40°C, the value of *F*_*p,max*_ was found to be *F*_*p*,*max*_≈0.45.

**FIGURE 3:**
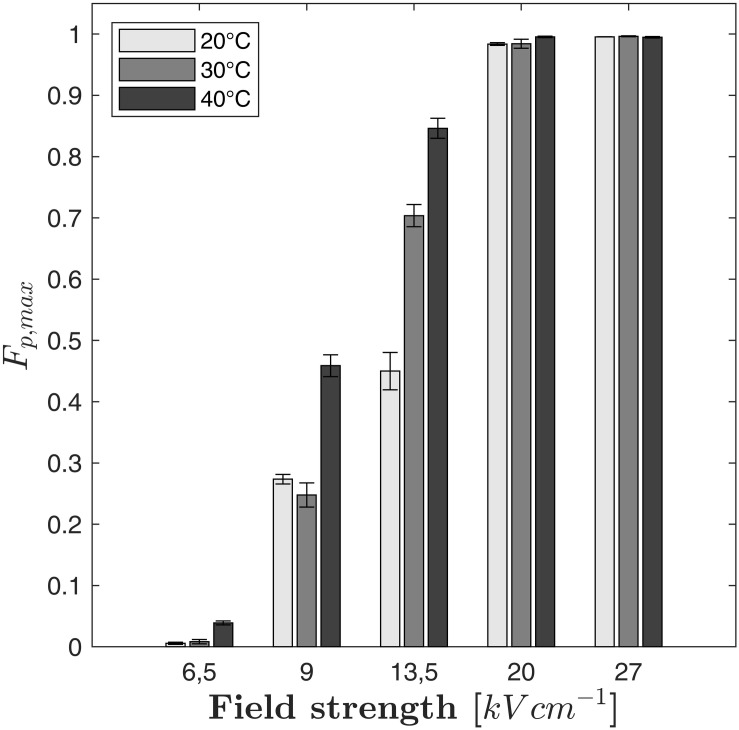
Measured maximum fraction of perforated cells (*F*_*p,max*_) with respect to the electric field strength and the treatment temperature. Error bars indicate the standard deviation.

As it can be seen in Figure 3, the maximum fraction of perforated cells *F*_*p,max*_ depends not only on the electric field strength but also on the treatment temperature. For example, *F*_*p,max*_ increased steadily with temperature at 13.5 kV cm^–1^ (*F*_*p,max*_ = 0.45 at 20°C, 0.704 at 30°C and 0.846 at 40°C). At 9 kV cm^–1^, a similar increase can be observed if the treatment temperature is changed from 30 to 40°C while no significant difference between treatments at 20°C and 30°C was found. If the electric field strength exceeds 13.5 kV cm^–1^, the value of *F*_*p,max*_ becomes independent from the treatment temperature because the entire cell population is electroporated. Regarding the modeling of the cell disruption kinetics, it is therefore crucial to describe *F*_*p,max*_ as a function of the electric field strength and the treatment temperature. However, the temperature plays an important role for the kinetics of cell perforation at all field strengths. As indicated by Figures 2A–C, the slope of the fitted curves increases with temperature at a given electric field strength (similar markers). Therefore, not only the parameter *F*_*p,max*_ is a function of temperature but also the time constant of the kinetic model.

The experimental results indicate that the cell permeabilization process can be realized at moderate temperature (e.g., 30°C) since a complete perforation of the cell population is achieved at a sufficiently high electric field strength. A further increase of temperature to 40°C only affects the number of required pulses to reach a complete permeabilization, thus the treatment time. This finding might be beneficial for the permeabilization of microalgae because less thermal energy must be spent for pre-heating of the suspension and in addition the risk of damaging thermally sensitive products can be reduced. The adequate description of the cellular permeabilization kinetics is therefore an important step for designing cell disruption processes and optimization of the operation conditions. Particularly, this is the case for PEF in colinear chambers due to the inhomogeneous electric field, velocity and temperature distributions, which lead to wide residence time distributions and local temperature hotspots (Gerlach et al., 2008; Jaeger et al., 2009; Wölken et al., 2017).

### Kinetic Model for Cell Perforation

#### Primary Model Equations

A suitable kinetic model for cell permeabilization must consider the electric field strength, the treatment temperature and the treatment time. The modeling approach applied in this work is based on the work of Saulis and Venslauskas (1993). In their model, the formation of pores in the cell membrane is considered as a Poisson process so that the fraction of non-perforated cells after time *t* is given by a Poisson distribution. A cell is assumed to be permeabilized if the number of pores exceeds a critical value. In the present study, cells are considered to be permeabilized as soon as the first pore appears because they become accessible for the PI staining. In that case, the Poisson distribution becomes a simple exponential distribution. As discussed above, the kinetic model must include an additional term *F*_*p,max*_ describing the upper limit for the fraction of perforated cells as a function of the electric field strength *E* and the treatment temperature *T*. With this extension, the kinetic model of Saulis and Venslauskas becomes

(9) $$F_{p}=F_{p,m\,a\,x}\,\left(1-\exp\left(-k_{f}\,t\right)\right)$$

Herein *k*_*f*_ is a kinetic parameter for the pore formation and *t* represents the treatment time. In the case of a batch treatment, it can be expressed as

$$t=n_{p}\,\tau$$

where τ represents the pulse duration and *n*_*p*_ the number of pulses, respectively. In the case of a continuous treatment chamber, the pulse repetition rate *f* and the residence time *t*_*R*_ needs to be taken into account (Raso et al., 2016). Therefore, *t* can be represented as

$$t=f\,\tau \,t_{R}$$

In order to derive the model parameters *k*_*f*_ and *F*_*p,max*_ as a function of the electric field strength and temperature, equation (9) was fitted to the experimental data shown in Figure 2. The built-in function *lsqnonlin* of MATLAB2019a was used for the fitting procedure, which minimizes a user-defined error function. For the present problem, the error was defined as the absolute deviation between the model and experimental measurements at constant temperature and electric field strength.

(10) $$\epsilon_{F\,p}=\sqrt{{\left(F_{p,S\,i\,m}-F_{p,E\,x\,p}\right)}^{2}}$$

*F*_*p,Exp*_ represents the experimental data and *F*_*p,Sim*_ the solution of equation (9). The iterated values of *k*_*f*_ and *F*_*p,max*_ were determined for all combinations of treatment temperature and electric field strength (*R*^2^ = 0.984), thus leading to a set of 15 values for each parameter. The root mean square error (RMSE) is used as a quality measure for the fitting procedure. The RMSE with dimension of *F*_*p*_ takes values of 0.0352 for 20°C, 0.0345 for 30°C, 0.0342 for 40°C and 0.0346 for the complete data set. The resulting fits are shown by the dashed lines in Figure 2. The determined parameters are named *k*_*f,exp*_ and *F*_*p*,*max*,*exp*_ hereafter.

#### Secondary Model Equations

**FIGURE 4:**
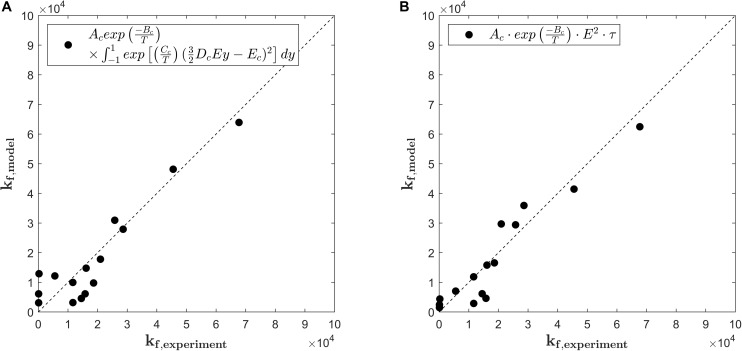
Parity plots for pore formation rate *k*_*f,experiment*_ obtained by a primary fit of the experimental data and the pore formation rate *k*_*f,model*_ as predicted by equation (11) **(A)** and equation (13) **(B)** with respect to the electric field strength *E* and total temperature *T*. The subscripted letter *c* in the legends indicates modeling constants.

In order to express the model parameters *k*_*f*_ and *F*_*p,max*_ in terms of the electric field strength and the temperature, secondary model equations are needed. Saulis and Venslauskas suggested a function which describes the rate of pore formation *k*_*f*_ by the product of an Arrhenius-type term for the temperature dependency of natural pore formation in the membrane and a second exponential term which accounts for the stabilization of naturally formed pores by the increase of the transmembrane potential. Since the transmembrane potential in an electric field is a function of the location on the cell surface (Krassowska and Filev, 2007), the latter is integrated over the cell membrane surface in order to calculate the overall effect of the electric field on the formation of pores. The proposed model for the rate of pore formation reads

$$k_{f}=A_{c}\left[s^{-1}\right]\exp\left(-\frac{B_{c}\,\left[K\right]}{T}\right)\times$$

(11) $$\int_{-1}^{1}e\,x\,p\,\left[\left(\frac{C_{c}\,\left[K\,V^{-1}\right]}{T}\right)\cdot {\left(\frac{3}{2}\,E^{2}\,D_{c}\,\left[m\right]\cdot {\text{y-E}}_{c}\,\left[V\right]\right)}^{2}\right]\,d\,y$$

where*A_c_*, *B*_*c*_, *C*_*c*_, *D*_*c*_, and *E*_*c*_ are constant fitting parameters summarizing different properties of the cell and the surrounding media. The reader is referred to Appendix A for their exact meaning. The quantity *y* is the substituent for *cos*⁡(ϕ), whereby ϕ is the angle to the cell surface normal. The best fit of equation (11) to the determined primary model parameter *k*_*f,exp*_ was calculated similarly as described above. The error function of the optimization was defined by the expression

(12) $$\epsilon_{k_{f}}=\sqrt{{\left(k_{f,m\,o\,d\,e\,l}-k_{f,e\,x\,p}\right)}^{2}}$$

where *k*_*f,exp*_ stands for the rate constants being determined by the fit of equation (9) to the experimental data and *k*_*f,model*_ for the solution of equation (11). Figure 4A compares the predicted values for *k*_*f,model*_ as calculated by equation (11) with the values of the fitting parameter *k*_*f,exp*_ of equation (9). The overall quality of the fit is indicated by the RMSE, which equals 6.428.10^3^ s^–1^. As it is indicated by the bisector, equation (11) with fitted parameters *A*_*c*_, *B*_*c*_, *C*_*c*_, *D*_*c*_, and *E*_*c*_ describes the general trends (*R*^2^ = 0.847) but fails to provide a detailed description of the rate constants at low to moderate treatment intensities. Even though the model for the rate of pore formation has a strong mechanistic background, its structure is complex and contains a large number of parameters, which entails the risk of overfitting. Moreover, regarding the intended implementation of the kinetic model into a holistic numerical model for the PEF process, equation (11) is inconvenient to solve and additional numerical costs arise through the need of integrating the transmembrane potential over the cell surface.

Therefore, a simplified model equation for the description of the pore formation rate *k*_*f*_ was developed and compared to the results obtained by equation (11). Sadik et al. (2014) showed for mouse fibroblast cells that the viability of the cells decreased linearly with the treatment time and quadratic with the electric field strength. According to these findings, *k*_*f*_ can be expressed as:

(13) $$k_{f}=A_{c}\,\exp\left(-\frac{B_{c}}{T}\right)\,E^{2}\,\tau$$

*A*_*c*_ is a fitting parameter which adjusts the influence of the electric energy and the temperature on the pore formation and takes the value *A*_*c*_ = 3.92.10^7^ V^2^ m^–2^s^–2^ for the present case. Similar to the model of Saulis and Venslauskas (1993), the temperature is taken into account by the Arrhenius model with parameter *B*_*c*_, which is determined as *B*_*c*_ = 7.28.10^3^ K^–1^. The overall quality of the fit is indicated by *R*^2^ = 0.895 and the RMSE = 5.687.10^3^ s^–1^, which is one order of magnitude smaller than the predicted values. As it can be seen from Figure 4B and the error measures, equation (13) predicts the values for *k*_*f*_ better than equation (11). Especially the low values of *k*_*f*_ are better described, which is particularly important with regard to the simulation of cell permeabilization under the inhomogeneous conditions in colinear continuous treatment chambers. Because of the higher accuracy and the structural benefits, equation (13) was chosen as a model for the rate constant *k*_*f*_.

The second parameter which needs to be expressed in terms of electric field strength and temperature is the maximum fraction of perforated cells *F*_*p,max*_. In order to obtain a secondary model equation, a two-step procedure is applied. The values being determined by fitting the primary model, equation (9), to the experimental data were fitted isothermally with a Weibull distribution as a function of the electric field strength:

(14) $$F_{p,m\,a\,x}=1-\exp{\left(-\frac{E}{\lambda_{S}}\right)}^{k_{S}}$$

The best fit of the parameters λ_*S*_ and *k*_*S*_ was obtained by finding the minimum of the error function:

(15) ϵ=Fp(Fp,m⁢a⁢x,m⁢o⁢d⁢e⁢l-Fp,m⁢a⁢x,e⁢x⁢p)2

**FIGURE 5:**
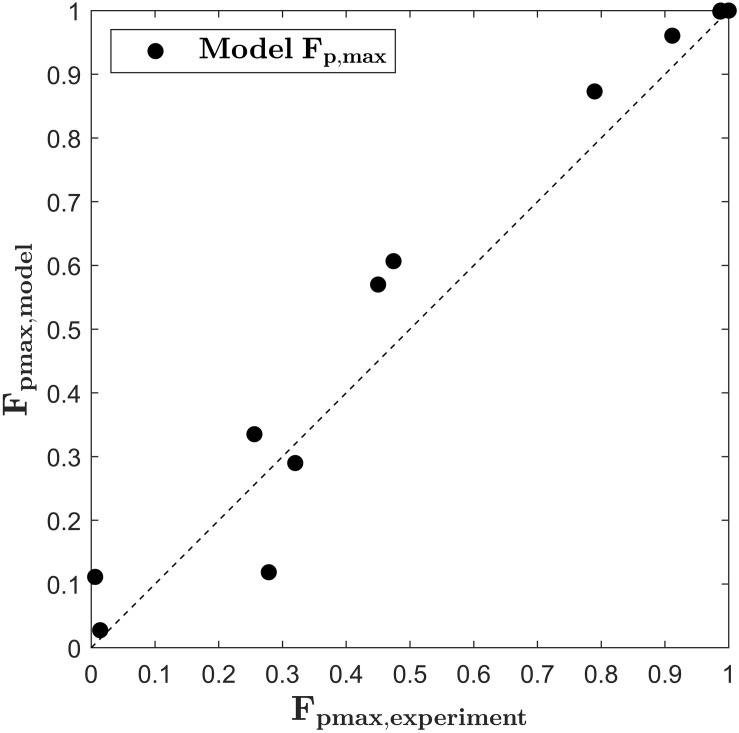
Parity plots for the maximum fraction of perforated cells (*F*_*p*,*max*_) according to equations (14)–(17) and *F*_*p,max*_ obtained by the primary fit of the experimental data.

Thereby, the value of *F*_*p,max*_ was restricted to the upper and lower bounds of 0 and 1. The quality of the fit is determined in terms of *R*^2^ = 0.977 and the RMSE = 0.057 in units of *F*_*p*_. In a second step, the fitted parameters λ_*S*_ and *k*_*S*_ were expressed in terms of the temperature with an exponential and a linear equation, respectively. The resulting equations read

(16) $$\lambda_{S}=A_{\lambda }\,\text{exp}\,\left(-B_{\lambda }\,T\right)$$

(17) $$k_{S}=A_{k}\,T+B_{k}$$

with the parameters *A*_λ_ = 6.683.10^9^ V m^–1^, *B*_λ_ = 0.0201 K^–1^ (*R*^2^ = 0.997), *A*_*k*_ = −0.0742 K^–1^ and *B*_*k*_ = 26.51 (*R*^2^ = 0.997). The quality of the fits is assessed in terms of the RMSE, which takes values of 4.786.10^4^ and 0.0131 in units of λ_*S*_ and *k*_*S*_, respectively, both being two magnitudes smaller than the predicted values. Figure 5 depicts the predicted values for *F*_*p*,*max*,*model*_versus the values of *F*_*p,max,exp*_. As indicated by the bisector, the developed model equation describes the general trends fairly good. At low electric field strength and low treatment temperatures, the model slightly overpredicts *F*_*p,max*_. Regarding the application of the model to the simulation of a continuous treatment chamber, this error seems of minor importance because *F*_*p,max*_ will probably not be reached during the common short residence times. For example, at 6.5 and 9 kV cm^–1^ 64 pulses were needed to reach *F*_*p,max*_. This number of pulses is very high for the case of a continuous treatment since the pulse repetition rate is often limited by the utilized processing equipment. In this study, a maximum of 22 pulses was applied in the colinear treatment chamber at the highest pulse repetition rate and the lowest flow rate, respectively (estimate is based on the mean residence time in the treatment zone). On the other hand, as it can be seen in Figure 5, one value is clearly underpredicted by the model. This value belongs to the treatment at 9 kV cm^–1^ and 20°C (compare also Figure 3). As described in section “PEF Treatment,” the experiments with the longest treatment time were conducted in triplicate and therefore it is unlikely that measurement errors explain the deviation. Instead, it might be caused by an erroneous estimate of *F*_*p,max*_ at these conditions. It can be seen from Figure 2, that the value of *F*_*p,max*_ might be slightly higher for treatments at low intensity since the maximum value was not reached after the longest treatment time. For future research it is therefore recommendable to choose the treatment time long enough for reaching a plateau for *F*_*p*_ at any treatment condition. For that, the control of the temperature during the PEF treatment is of high importance in order to distinguish between PEF and temperature effects. Nevertheless, the aim of the study is to find optimal parameters for the upscaling of the treatment chamber so that treatment conditions leading to low degrees of permeabilization are of minor interest.

#### Overall Kinetic Model

For the usage of the cell permeabilization kinetics in the process model (see section “Governing Equations”), a rate equation for the perforation of cells must be formulated, which represents the source term π_*F_p*_ in equation (7). The source term represents the fraction of intact cells being permeabilized per time increment. Therefore, π_*F_p*_is given by the time derivative of equation (9), which is

(18) $$\pi_{{\text{F}}_{\text{p}}}=\frac{d\,F_{p}}{d\,t}=k_{f}\,\tau \,f\,\left(F_{p,m\,a\,x}-F_{p}\right)$$

**FIGURE 6:**
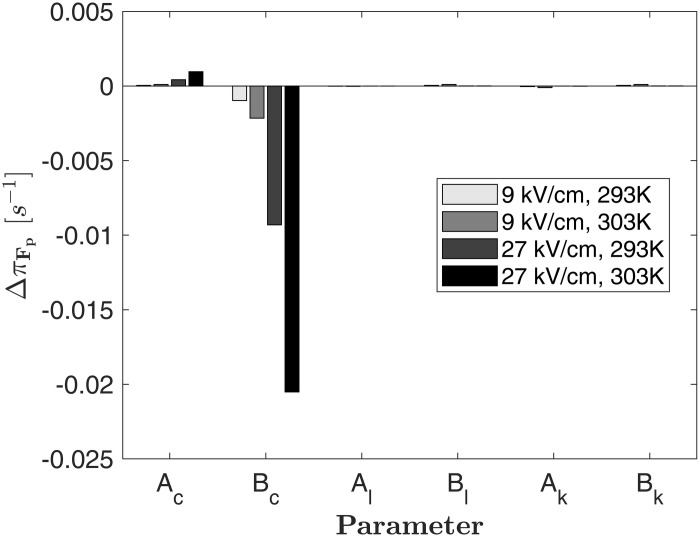
Parameter sensitivity of the source term as given by equation (18) for *F*_*p*_ = 0 and different values of the temperature and electric field strength. The quantity Δπ_Fp_ is the change of the source term if one parameter is increased by 1% of its value while all others are kept constant.

The parameters in equation (18) need to be determined by equations (13), (14), (16) and (17). Figure 6 shows the local parameter sensitivity of π_*F_p*_, which is determined by measuring the change of the model output Δπ_Fp_ if one of the 6 parameters is increased by 1% of its value, while all other parameters are kept constant (Hamby, 1994). Assuming *F*_*p*_ =  0 and different values for *E* and *T*, the highest sensitivity of the model is found for changes of the parameters *A*_*c*_, and *B*_*c*_, which relate the reaction rate constant to *E* and *T*. This result reflects the structure of equation (13) and the respective linear or exponential impact of the parameters. If *F*_*p*_ is increased, the sensitivity of the model to *A*_*c*_, and *B*_*c*_ decreases linearly but the model remains most sensitive toward *A*_*c*_, and *B*_*c*_, which have therefore the largest impact on the source term.

### Validation of the Kinetic Model

**FIGURE 7:**
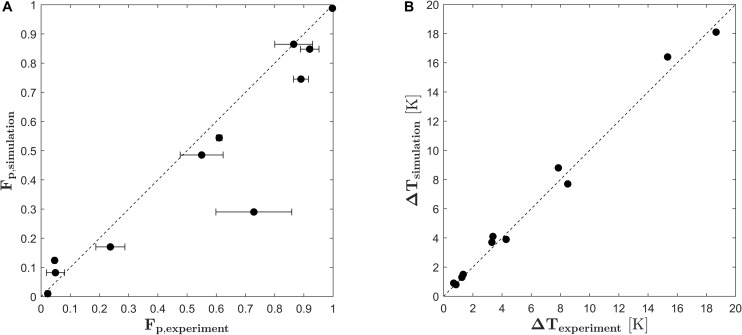
Parity plots of the fraction of perforated cells *F*_*p*_ **(A)** and the average temperature increase **(B)** in the validation experiment versus the results obtained by CFD simulation. Error bars indicate the standard deviation of the experimental runs.

In order to validate the PEF process model and the derived kinetic model for cell electroporation, experiments were conducted in a pilot scale colinear treatment chamber and compared to the simulation results of the similar treatment plant. Therefore, equation (18) was implemented into the finite volume code Ansys CFX19 together with equations (13), (14), (16) and (17) and used to calculate the degree of cell disruption in a colinear treatment chamber by solving equations (1)–(7). The results for the fraction of perforated cells obtained in the validation experiment are plotted in Figure 7A against the results obtained by the CFD simulations. As being indicated by the angle bisector, the simulated cell permeabilization is in good agreement to the experiments. Some deviations can be observed for the inlet temperature of 30°C and the highest specific energy inputs (for example *F*_*p,exp*_ = 0.9 and *F*_*p*,*sim*_ = 0.75). Here, the model underpredicts the experimental results. One possible explanation is that the average temperature increase for this condition was larger than 10 degrees and therefore, the outlet temperature was almost at 50°C. Since this is beyond the calibrated range of the temperature (20–40°C), the enhancement of the PEF effect through the higher temperature might be underestimated for these conditions. Furthermore, the critical temperature for thermally induced autolysis of *Chlorella vulgaris* was observed to be 55°C (Postma et al., 2016). At high treatment intensities, these temperatures can be reached or even exceeded locally within the chamber so that an additional thermal effect on the cell viability might be possible, which however is not included in the presented model. Figure 7A shows also that one point clearly deviates from the experimental data. Because the simulated result is in line with the remaining data and also the predicted temperature increase matches the experiment (see Figure 7B), it is likely that the deviation between simulated and measured cell permeabilization is caused by an experimental error, which is also indicated by the large standard deviation of this data point.

The general validity of the PEF process model was tested by comparing the predicted temperature increase in the treatment chamber with the measured data. The results are plotted in Figure 7B. Again, the simulation results agree in a good way with the experimental data, which means that the specific energy input in the simulation matches the specific energy input in the experiments.

In conclusion, one can say that the good agreement between the results of the CFD simulation and the experimental data in a colinear treatment chamber shows the validity of the derived kinetic model. It should be emphasized that the kinetic model was calibrated with data being obtained in batch experiments in laboratory electroporation cuvettes. On the contrary, the model validation was not only performed on the larger pilot scale but also in a continuous treatment chamber, for which the occurrence of inhomogeneous treatment conditions is well known. Therefore, the study demonstrates the potential of the proposed approach as a method to transfer data from a small to a larger scale. Since the model is independent of the investigated treatment chamber design, it can be used to design and optimize new treatment chambers for the treatment of microalgae.

### Results of the Numerical Parameter Study

After validation, the PEF process model can be applied to study the effect of different process conditions on the degree of cell permeabilization. For that purpose, a numerical parameter study is conducted as described in section “Numerical Parameter Study.”

**FIGURE 8:**
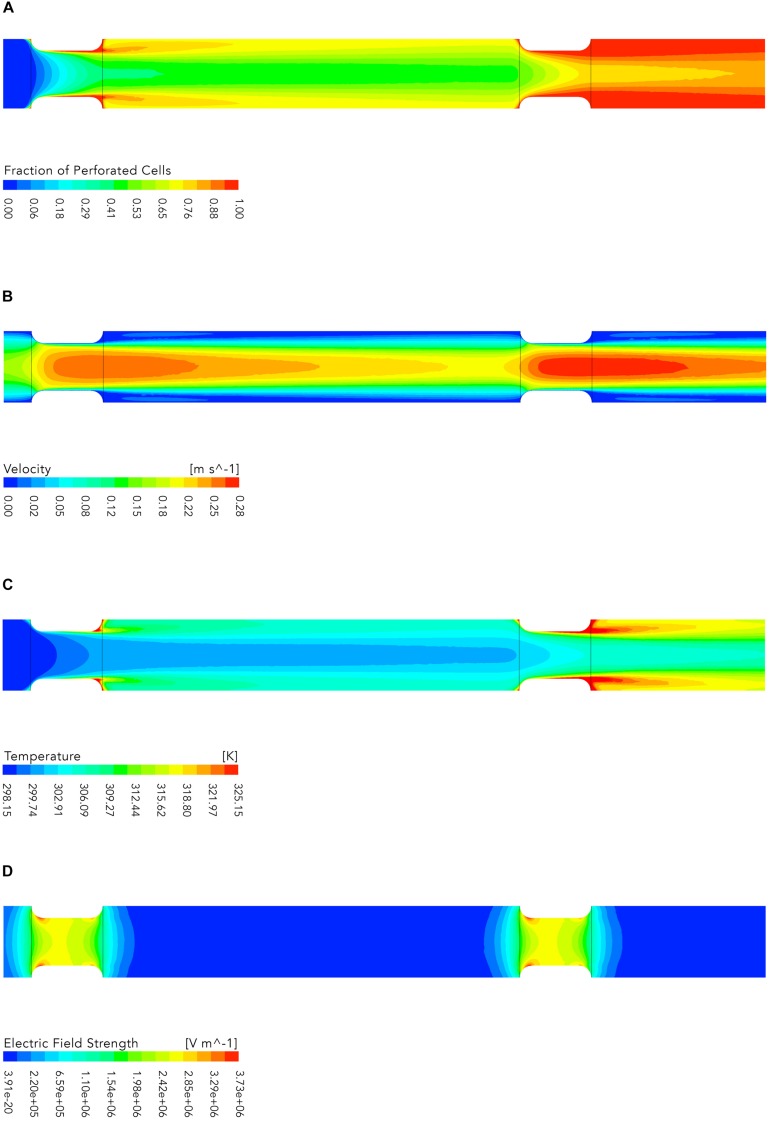
Contour plots of the fraction of perforated cells **(A)**, flow velocity **(B)**, temperature **(C)**, and electric field strength **(D)**, respectively. The plots show the middle plane of the colinear treatment chamber. The flow is from left to right. The electric energy input *w*_*spec*_ for the shown case was 58.62 *kJkg*^−1^ (*U* = 15*kV*, *T*_*I*_ = 298.15*K*, $\dot{V}=2.50\cdot 10^{-6}m^{-3}s^{-1}).$ Note that the plots depict not the entire simulated domain (see Figure 1), since the parts not shown contain no additional relevant information.

The results of the numerical simulations are exemplary shown in Figure 8 for one of the simulated cases (*U* = 15kV, *T*_*I*_ = 298.15K, $\dot{V}=2.50\cdot 10^{6}{\text{m}}^{-3}s^{-1}).$Figure 8A shows the evolution of the fraction of perforated cells *F*_*p*_ in the treatment chamber. In the first treatment zone about 45% of all cells are permeabilized and the highest inactivation occurs in regions close to the wall where the flow velocity is low. In addition, a high fraction of permeabilized cells can be found behind the insulators where cells are trapped in recirculation zones with high temperature (see Figures 8B,C). The second plot shows the flow velocity magnitude, which follows the typical patterns of laminar flow with the lowest magnitudes near the wall of the chamber and the largest in the center of the pipe. This corresponds to a shorter treatment time for cells which are passing the treatment zone in the middle of the insulators. Therefore, it is obvious that the inactivation is lower in the center of the treatment chamber. This effect is further enhanced because the electric field strength is stronger near the insulator walls (see Figure 8D).

The specific energy input is of major interest for PEF treatment. It can be calculated per kg of cell suspension by integrating the local energy input over the volume of the treatment chamber, which yields the expression

(19) $$w_{s\,p\,e\,c}=\frac{\tau \,f}{\dot{m}}\,\int_{V}\sigma \,\left(T\right)\,E\,d\,V$$

Herein, $\dot{m}$ is the mass flow rate of the liquid and *V* the volume the chamber. Since preheating contributes to the overall energy demand, the total energy input *w*_*total*_ should be calculated as

(20) $$w_{T\,o\,t\,a\,l}=w_{s\,p\,e\,c}+c_{p}\,\text{Δ}\,T$$

**FIGURE 9:**
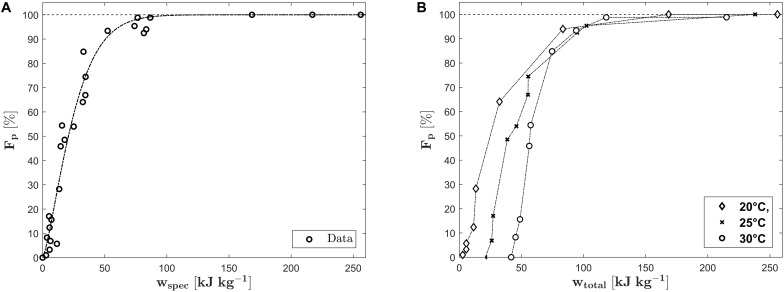
Fraction of perforated cells (*F*_*p*_) as a function of the electric energy input *w*_*spec*_ **(A)**. The dash dotted line indicates the best fit of the data by the Weibull distribution function. Plot **(B)** shows the fraction of perforated cells versus the total energy input *w*_*total*_.

Herein stands Δ*T* for the temperature difference between a reference temperature (in this case chosen as 20°C) and the actual treatment temperature at the chamber inlet. An average value for the heat capacity was used for the calculation of the energy demand for preheating of the cell suspension. Figure 9A depicts the fraction of perforated cells versus the electric energy input. For the complete permeabilization of the cell suspension, an energy input of at least 64.64 kJ kg^–1^ is necessary. This is around three times more than the energy input which was necessary for a full perforation in the batch experiments (see section “Experiments in Electroporation Cuvettes”). This dramatic increase can be explained by the previously discussed inhomogeneous treatment conditions in the chamber. Furthermore, the residence time within the treatment zone inside the two isolators is short so that the number of applicable pulses is limited. For the treatment chamber under investigation, mean residence times of 0.062 s, 0.0417 s and 0.0313 s were calculated for volume flows of 1.66 ml s^–1^, 2.5 ml s^–1^ and 3.33 ml s^–1^, respectively. This corresponds to a maximum of 22 pulses on average at the lowest flow rate and the highest pulse repetition rate (200 Hz). Due to limitations of the used equipment, the maximum applicable electric field strength is 22.8 kV cm^–1^. Regarding the results of section “Experiments in Electroporation Cuvettes,” it seems likely that the required specific energy input for permeabilizing all cells of *Chlorella vulgaris* could be reduced by applying higher voltages. However, the example makes clear that the design of the treatment chamber is fundamental for energy efficient processing and further developments in this direction are needed.

The variance of the data in Figure 9A can be explained by the different treatment temperatures. If the data are plotted with respect to the total energy input, the variance within each group almost disappears (see Figure 9B). It can be observed that the overall energy input for a full perforation is more or less similar for all inlet temperatures. Therefore, preheating might be a good option to improve the cell permeabilization in case of technical limitations of the pulse generator. On the other hand, it was stated before that the extraction of thermosensitive cell valuables might be the target of the process and that it might be favorable to choose a lower inlet temperature to remain their functionality. Nevertheless, the temperature increase for a full perforation at an inlet temperature of 20°C was 31°C (*T*_*outlet*_ = 51°C), 28.87°C for an inlet temperature of 25°C (To⁢u⁢t⁢l⁢e⁢t=53.87C∘) and only 16.11°C for an inlet temperature of 30°C (To⁢u⁢t⁢l⁢e⁢t=46.1C∘). The results show that finding optimal conditions is not always straightforward and that numerical simulation can be a tool to support the process design.

## Discussion

### Discussion of Experiments in Electroporation Cuvettes

During the treatment of microalgae cells in electroporation cuvettes, it was observed that the maximum achievable fraction of permeabilized cells depends on the applied treatment conditions. An explanation for the observed maximum degree of cell permeabilization is the dependency of the transmembrane potential on the cell size (Neumann, 1996). Even if the mechanism of pore formation is not fully understood, it is consensus that a critical transmembrane potential must be exceeded to induce pore formation (Zimmermann et al., 1976; Abidor et al., 1979). Because the size of cells within a population is never unique but follows a size distribution, a simple explanation for the observed results could be that the critical transmembrane potential was not exceeded for a certain proportion of the cell population. The cell size distribution was also measured for some samples during the experiments and the obtained results indicate a correlation between 1−*F*_*p*,*max*_ and the fraction of the cell population with size larger than a critical value. However, the results are preliminary and further experiments must be carried out to substantiate the hypothesis that incomplete cell perforation is linked to the cell size distribution under certain treatment conditions. The observed phenomena of converging to a maximum value for the fraction of perforated cells under certain conditions is in good agreement to the data of Martínez et al. (2019). They investigated the permeability of *Porphyridium cruentum* cells to PI as a function of electric field strength and the treatment time. In this work it was also shown that the increase of the treatment time did not increase the number of PI-stained cells at certain treatment conditions.

At the lowest treatment intensities, the maximum degree of cell permeabilization was almost not measurable. It is possible that a larger number of cells was permeabilized under these conditions but that resealing of the pores took place before the fluorescent dye was added. For example, Luengo et al. (2014) showed that the addition of PI before the PEF treatment led to a higher proportion of stained cells in comparison to the addition of the dye after the treatment. This means that even at a low electric field strength, a certain part of the cells can be reversibly perforated and therefore becomes accessible for PI, which might be interesting for cell milking applications (Buchmann et al., 2019). However, the concept of microalgae milking has not been proven yet to work at relevant scale and PEF treatment is usually applied to reduce the mass transfer resistance of cells in order to improve the extraction of valuable cell contents.

Resealing is not the only mechanism which affects the accessibility of fluorescent dyes like PI to cells after PEF. Kennedy et al. (2008) observed a rise in the fluorescence intensity over up to 500 s after the treatment and PI addition. Similar results were found by Sweeney et al. (2018b) who observed especially at low treatment intensities an increasing fluorescence intensity for a certain fraction of the treated cells during a time interval of more than 5 min after the treatment and PI addition. The authors attribute their observations to a number of possible reasons, namely diffusive transport of PI into the cells, the formation dynamics of the PI-nucleic acid-complex in a cell and the dynamic closure (Kennedy et al., 2008) or formation of pores after the treatment (Sweeney et al., 2018b). For the development of kinetic models, it is therefore important to use accurate staining protocols, which ensure the correct detection of the permeabilized cell fraction, even at low treatment intensities. The applied experimental protocol for the PI staining of *C. vulgaris* was optimized by Design of Experiments with the independent variables PI concentration per biomass, temperature and time. Since the time duration between the treatment and the measurement in the flow cytometer was about 30 min, it can be expected on basis of the available knowledge that the electroporated fraction of the cell culture remains stable for a suitable time interval and therefore that the measured data provides reliable information about the impact of different treatment conditions on the fraction of permeabilized cells.

### Discussion of Kinetic Modeling and Numerical Simulation as Tools for Electroporation Process Development and Scale-Up

The good agreement between the simulation results and the performed experiments in a colinear treatment chamber show the validity of the developed process model. This further proves that simulation is a valid tool to transfer data from a small cuvette scale to a larger scale, even if the design of the treatment chamber and the process operation mode change. Therefore, modeling and simulation can be considered as important tools for the process scale-up or for the design of new treatment chambers for PEF processing. This opens the possibility to develop tailored geometries for the treatment of microalgae with a high throughput at the industrial scale.

For the scale-up it is important to consider process homogeneity and energy requirements, which also depend on the configuration of the treatment chamber. The presented experimental results indicate that a minimum specific energy input of 19.6 kJ kg^–1^ is necessary to perforate 100% of the cells in electroporation cuvettes. These results are in good agreement with the work of Rego et al. (2015), who treated *Chlorella* cells in a continuous treatment chamber with a parallel plate electrode configuration. According to their results, about 20 kJ kg^–1^ were necessary for permeabilizing 100% of the treated cells. In this case, the good comparability of a batch and a continuous treatment chamber may be due to the parallel plate arrangement, which however is not suitable for industrial applications since this configuration has a lower electrical resistance and therefore a high current flow. Therefore, higher energy inputs are necessary to reach the required electric field strength in a treatment chamber with a parallel plate configuration (Jaeger et al., 2009). According to the presented simulation results, at least 64.64 kJ kg^–1^ are necessary to achieve the complete perforation of the cell culture in the investigated colinear arrangement. By the best knowledge of the authors, no work was published which investigates the treatment of *Chlorella vulgaris* in a colinear treatment chamber at pilot scale so that it is not possible to compare the measured data with the literature. However, the mentioned discrepancy between different setups at different scales shows on the one hand that numerical simulation can serve as a useful tool for the development and planning of industrial biorefinery concepts because estimates of the required energy input and process conditions can be achieved *a priori*. On the other hand, the energy requirement for treating microalgae can be potentially reduced and additional work is needed to study microalgae cell permeabilization at scales beyond the lab scale and to develop energy efficient treatment chambers. This is particularly of interest since the energy input scales linearly with temperature increase. The presented simulation results for different PEF treatment conditions indicate a large effect of the local temperature magnitude and the flow field on the cell membrane permeabilization of *Chlorella vulgaris*. If mass transfer enhancement for the extraction of valuable compounds is the goal of the process, long residence times and the exposure to high temperatures may lead to a loss of thermally sensitive products which in turn entails a lower economic efficiency. It is therefore questionable whether a colinear treatment chamber is the best option for treating microalgae. Numerical simulation and validated virtual engineering are suitable tools to develop new treatment chambers which avoid the occurrence of temperature hotspots. According to the presented results, the improvement of the flow field might be a good starting point to create new treatment chambers for the electroporation of microalgae.

Nevertheless, several practical aspects need to be considered when models like the proposed one shall be used for process or plant design. First, one should consider the pulse shape in the real process. Since the impedance of the treatment chamber might not fit to the pulse generator, reflection can occur. Therefore, the real pulse applied to the suspension might be different from the perfect rectangular one which is considered in the simulation. As stated in section “PEF Systems and Experimental Design,” an overshoot of the voltage and subsequent ringing was observed during the conducted experiments. These features of the pulse are not resolved by the numerical model. Instead, it considers the time-averaged power input, which is the power input per pulse being scaled with the pulse repetition rate, see equation (4) and definitions in section “Governing Equations.” The comparison between experiments and CFD simulation indicates that the most important factors for the prediction of cell permeabilization (Figure 7A) and specific energy input (Figure 7B) are correct values for the time-averaged voltage during a single pulse and the energy per pulse under the investigated conditions. It can be expected that this conclusion is valid up to a certain limit but also that the numerical model does not reflect the real conditions anymore if the difference between real and perfect pulses becomes too large. Consequently, the prediction of cell permeabilization will also deviate from the experimental reality.

Second, the results of this study indicate that thermally induced autolysis might take place in real processing. For *Chlorella vulgaris* this is the case at temperatures higher than 55°C (Postma et al., 2016). Since such effects are not considered in the proposed model, thermal autolysis can explain deviations to experiments if the inlet temperature or the electric energy input are too high.

Third, the dependency of the PEF response on the growth phase is known for a number of other organisms such as *Saccharomyces cerevisiae* (Molinari et al., 2004). It is thinkable that the time of cell harvest and the respective growth phase have an impact on the predictive capability of the developed kinetic model. In that case it would be crucial for a successful process design to know the harvesting point and the constitution of a cell culture before calibrating kinetic models for cell perforation. In the present work, cells were harvested in the late exponentially phase, what should be considered if the proposed model is used by other scientists or process developers.

Fourth, it should be mentioned that the model calibration was done at a dry mass content of 1 g l^–1^. Nevertheless, it was shown that the PEF treatment of microalgae is independent of the biomass concentration up to concentrations 160 g l^–1^ for Auxenochlorella protothecoides (Goettel et al., 2013) and 40 g l^–1^ for Chlorella vulgaris (Rego et al., 2015) (higher concentrations were not tested in the cited papers). Based on these results, the validity of the proposed model at higher biomass concentrations seems likely, although it should be proved in future work. Also, it must be shown whether this assumption is valid for dry mass concentration higher than 40 g l^–1^, which are of interest for industrial applications because the specific energy input per unit of dry biomass decreases at higher biomass concentrations. Even if the cell concentration has no direct impact on the perforation kinetics, it should be considered since the effective viscosity and the flow behavior of the cell suspension changes at higher biomass concentration. This entails an indirect effect on the degree of perforated cells, due to its dependency on the residence time distribution, which again is affected by the suspension rheology. Even if this point can be addressed easily in simulations with suitable data for the concentration dependency of the viscosity at hand (Buchmann et al., 2018), it should be kept in mind in the case that the cell concentration is changed.

Lastly, the development of kinetic models requires a method for the detection of cell permeabilization. In this work, PI staining and flow cytometry were the chosen for this task. With these methods, the detection of cells in which the fluorescent dye has been penetrated is an all or nothing event and therefore no statement regarding the degree of perforation of individual cells can be made. In other words: after a sufficient staining time, the method provides information whether a cell is permeabilized, but it is not suitable to give an answer to the question how much a cell is permeabilized. As discussed in the previous section, the uptake kinetics of PI are determined by the permeabilized fraction of the cell surface and its evolution in time (Kennedy et al., 2008). It can be expected that similar dependencies exist for the molecular mass transfer of other molecules than PI. Since the enhancement of solute mass transfer is the major application of PEF in the context of microalgae biorefinery, information about the relation between PEF treatment conditions and the consequential mass transfer kinetics might be of interest. Modeling and simulation turn out to be suitable tools for this purpose as well. In literature, authors suggest different approaches for relating the treatment conditions directly to the transport of solutes, which represents an extension in comparison to the presented model. Thereby, concentration gradients as driving forces (Mahnič-Kalamiza et al., 2014) and the transport resistance must be considered. The latter is related to the dynamics of pore formation and their size distribution (Krassowska and Filev, 2007; Sweeney et al., 2018a), which in turn determine the membrane permeability for specific solutes. An alternative approach to capture this effect is the introduction of a solute-specific hindering coefficient (Mahnič-Kalamiza et al., 2014). Even if these approaches are not formulated specifically for microalgae, the underlying concepts seem well applicable and provide beneficial approaches for future work in the field of utilizing PEF for biorefinery concepts. Nevertheless, new studies also show that the mass transfer is further enhanced by simply incubating the permeabilized cells after the PEF treatment. The associated decrease of the transport resistance is believed to be caused by an enzymatically driven autolysis in response to PEF-induced cell death (Silve et al., 2018a; Jaeschke et al., 2019; Scherer et al., 2019). Therefore, effects on short and on long time scales should be considered for the goal of improving the efficiency of microalgae biorefining by applying PEF technology.

## Conclusion

The broad commercial breakthrough of microalgae as a resource for versatile products in the food and feed industry and in industrial biotechnology is limited by the production costs. Therefore, it is crucial to find new cost and energy saving technologies for the downstream processing. The presented work proposes an approach for this objective based on modeling and numerical simulation of cell permeabilization by Pulsed Electric Fields. Simulations are highly desirable because they can contribute to reduce costs for the collection of data being required for process scale-up. The proposed approach follows the idea that data for the calibration of models for cell disruption kinetics can be gained in laboratory work at the smallest scale in electroporation cuvettes. Once the kinetic is adequately described, numerical simulation models offer flexible tools for process design, scale-up and optimization of operation conditions. The principal capability of this approach was demonstrated by comparing the results of simulations and experiments for the PEF treatment of *Chlorella vulgaris* in a pilot scale collinear treatment chamber. Therefore, the present work provides the first contribution showing the applicability of kinetic modeling and numerical simulation for designing PEF processes for the purpose of biorefining microalgae biomass.

Future work should target the enhancement of the treatment efficiency by designing new treatment chambers. With the proposed modeling approach, new designs can be compared easily with regard to their potential for energy efficient cell membrane permeabilization. In the end, numerical simulation cannot replace the manufacturing of prototypes, but it can help to find efficient designs at reduced cost for the process development.

## Data Availability Statement

The datasets generated for this study are available on request to the corresponding author.

## Author Contributions

JK and CM drafted the manuscript. JK conducted the experiments, analyzed experimental and simulation data, calibrated and implemented the kinetic model, and performed numerical simulation. CM conceptualized the study and supervised the experimental and numerical work including data analysis and model development. CR critically revised the manuscript, improved the work with important intellectual content, and took responsibility for the integrity of the study as a whole. All authors read and approved the submitted manuscript.

## Conflict of Interest

The authors declare that the research was conducted in the absence of any commercial or financial relationships that could be construed as a potential conflict of interest.

## Appendix A

The biophysical meaning of the parameters in equation (11) shall be briefly described. For the reader’s convenience, the equation is rewritten.

(21) $$k_{f}=A_{c}\,\left[s^{-1}\right]\,\exp\left(-\frac{B_{c}\,\left[K\right]}{T}\right)\times \int_{-1}^{1}e\,x\,p\,\left[\left(\frac{C_{c}\,\left[K\,V^{-1}\right]}{T}\right)\cdot {\left(\frac{3}{2}\,E^{2}\,D_{c}\,\left[m\right]\cdot {\text{y-E}}_{c}\,\left[V\right]\right)}^{2}\right]\,d\,y$$

In order to reduce the parameters of the original model as stated by Saulis (2010), the following substitutions were made

(22) $$A_{c}=\frac{2\,\pi \,\nu \,a^{2}}{a_{l}}$$

(23) $$B_{c}=\frac{\text{Δ}\,W_{f}\,\left(0\right)}{k_{B}}$$

(24) $$C_{c}=\frac{\pi \,C_{m}\,\left(\frac{\varepsilon_{W}}{\varepsilon_{M}}-1\right)}{2\,k_{B}}\,r_{*}^{2}$$

(25) $$D_{c}=a\,y$$

(26) $$E_{C}={\text{Φ}}_{0}$$

The symbols in Equations (22–26) have the following meaning: ν is the natural fluctuation frequency of the lipid molecules in the cell membrane, *a* is the cell radius, *a*_*l*_ the area of a lipid molecule, Δ*W*_*f*_(0) is the energy barrier for pore formation at a transmembrane potential of 0 V, *k*_*B*_ is the Boltzmann constant, *C*_*m*_ is the cell membrane capacity, ε_*W*_ and ε_*M*_ stand for the relative permittivity of water and the cell membrane, *r*_***_ is the radius of a pore corresponding to the maximum of the energy barrier at given transmembrane potential, *y* is the substitute for the integration variable which reads as cos(ρ) where ρ is the angle to the surface normal and Φ_0_ is the resting potential of the cell.
